# Poor cough flow in acute stroke patients is associated with reduced functional residual capacity and low cough inspired volume

**DOI:** 10.1136/bmjresp-2017-000230

**Published:** 2017-10-26

**Authors:** Katie Ward, Prashant Rao, Charles C Reilly, Gerrard Francis Rafferty, Michael I Polkey, Lalit Kalra, John Moxham

**Affiliations:** 1 Division of Asthma Allergy and Lung Biology, Respiratory Medicine, King’s College London, London, London, UK; 2 Respiratory Medicine, Royal Free Hospital, London, London, UK; 3 Department of Medicine, University of Arizona, Tucson, Arizona, USA; 4 Department of Child Health, Kings College London School of Medicine, London, UK; 5 Respiratory Medicine, NIHR Biomedical Research Unit at the Royal Brompton and Harefield Foundation Trust, London, UK; 6 Imperial College London, London, UK; 7 Department of Neurosciences, Academic Neurosciences Centre, King’s College London, London, UK

**Keywords:** cough/mechanisms/pharmacology, respiratory measurement, respiratory muscles, lung physiology

## Abstract

**Introduction:**

Each year 7 million people die of stroke worldwide; most deaths are caused by chest infections. Patients with acute stroke have impaired voluntary cough flow, associated with increased risk of chest infections. Reduced functional residual capacity (FRC) could lead to impaired cough flow. We therefore compared FRC in acute hemiparetic stroke patients and controls and explored its relationship with volume inspired before cough and voluntary cough peak flow.

**Methods:**

21 patients within 2 weeks of first-ever middle cerebral artery territory (MCA) infarct (mean (SD) age 68 (11) years, 10 females) and 30 controls (58 (11) years, 15 females) underwent FRC and voluntary cough testing (cough inspired volume and peak flow) while semirecumbent. FRC was expressed as % predicted; cough inspired volume was expressed as % predicted VC and cough peak flow as % predicted PEF. A clinician scored stroke severity using the National Institutes of Health Stroke Scale (NIHSS).

**Results:**

Patients’ reclined FRC, voluntary cough peak flowand cough inspired volume were reduced compared with controls (p<0.01 for all): patients’ median (IQR) FRC 76 (67–90) % predicted, mean (SD) cough inspired volume 64 (20) % predicted and mean (SD) peak cough flow 61 (32) % predicted despite them having only mild stroke-related impairments: median NIHSS score 4 (IQR 2–6). Univariate linear regression analyses showed FRC predicted cough inspired volume (adjusted R^2^=0.45) and cough inspired volume predicted cough flow (adjusted R^2^=0.56); p<0.01 for both. Sitting patients upright increased their FRC by median 0.210 L.

**Conclusions:**

FRC and cough inspired volume in the reclined position are significantly reduced in acute hemiparetic stroke patients with mild impairments; both factors are associated with poor voluntary cough peak flow.

**Key messages**

- Stroke patients have impaired voluntary and reflex cough flow which is associated with the development of chest infections.
- This study found that FRC (% predicted) is significantly reduced in acute hemiparetic stroke patients and this is associated with poor cough flow.
- Interventions which increase FRC deserve evaluation in MCA stroke patients at risk of chest infection.

## Background

Every year 17 million people experience their first stroke and 7 million will die of stroke worldwide. There are 1.2 million stroke survivors in the UK alone.^1^ Most stroke deaths are caused by complications, usually chest infections. Giving prophylactic antibiotics to stroke patients with unsafe swallow did not reduce incidence of poststroke pneumonia in a recently published, large multicentre study. Understanding impaired cough physiology post stroke is very important as we seek to reduce respiratory morbidity and mortality in this large patient group.^2^

Cough can help clear aspirated material from the lungs; weak (or absent) reflex and voluntary cough in stroke patients are both significantly associated with development of chest infections.^3–5^ Acute hemiparetic stroke patients have impaired voluntary and reflex cough flows^4 6–8^ and the more severe the patient’s stroke, the worse the cough flow.^6^ Reduced functional residual capacity (FRC) could cause impaired cough flows due to impaired flow volume characteristics of the deflating lungs and due to the effect of reduced lung volume on the length and thus the pressure-generating capacity of the expiratory muscles.^9^

In a previously published study of acute stroke patients, we showed that three predictors (stroke severity score, height and FEV_1_/FVC ratio) accounted for two-thirds of the variability in voluntary cough flow.^6^ We concluded that there must be additional factors implicated in variability of cough flow after stroke and one factor could be FRC. It is known that the volume of air in the lungs, immediately prior to an expulsive man oeuvre, has a linear relationship with the flow.^10^

Previous data on FRC in acute stroke are not available. There are very few published data on FRC in stroke patients, probably due to the difficulty of making measurements in this group. FRC could become reduced as a consequence of previously described chest abnormalities in stroke patients such as elevation of the diaphragm on the paretic side^11 12^; reduced movement of the diaphragm during inspiration^13^; weak respiratory muscles^8 14 15^; impaired corticofugal pathways to the diaphragm^16 17^ or flattening or reduced movement of the chest wall on the weak side.^18–20^ Stroke patients can spend a lot of time in reclined positions so the effect of posture on lung volumes is also relevant. FRC has been shown to fall by between 20% and 29% when healthy participants move from the seated to supine position.^21 22^

In this study, we evaluated FRC in acute hemiparetic stroke patients, with the objective to measure FRC and cough flow in acute stroke patients and compare them with healthy controls, as well as investigate the relationship between FRC and cough flow.

The null hypothesis was that FRC, measured by helium dilution in the reclined position, would be the same for patients with stroke as for a group of healthy controls. As a secondary hypothesis we sought to confirm that stroke patients have impaired voluntary cough flow and reduced air volume inspired prior to cough (cough inspired volume) compared with controls.^6^ These measurements would enable exploration of the relationship between FRC, cough flow and cough inspired volume. We also decided to measure upright FRC in a subgroup, to look at the effect of position change.

## Methods

### Study subjects

Patients were recruited from the Acute Stroke Unit of King’s College Hospital. Seventy-seven patients admitted within 2 weeks of middle cerebral artery territory infarcts were screened. Patients unable to follow verbal commands were excluded (n=13) as were those unable to use a snorkel-type mouthpiece correctly while wearing a nose clip (n=12). Others were excluded because of previous stroke (n=8) or respiratory disease (n=15). A total of 27 participants were suitable and consented. A group of 30 healthy controls were recruited from among hospital volunteers and laboratory staff.

### Baseline assessments

Smoking history, self-reported ethnicity, height and weight were recorded for all participants. For patients, stroke diagnosis was made by a consultant physician and confirmed radiologically. Stroke location was obtained from CT or MRI scan reports made by a consultant neuroradiologist. Stroke patients who met the thrombolysis eligibility criteria had received alteplase treatment at the time of admission.^23^ Stroke severity on admission and on the day of testing for this study was assessed using the National Institutes of Health Stroke Scale (NIHSS) by a stroke specialist doctor.^24^ The maximum score is 42; strokes scoring >25 are described as very severe and those scoring 1–5 are mild.^24^ A score of ≥16 forecasts a high probability of death or severe disability, whereas a score of ≤6 forecasts a good recovery.^25^ For further information on the NIHSS, see online supplementary file 1. Pulse oximetry was performed at rest in the reclined position, breathing room air (Ohmeda Biox 3740 Pulse Oximeter, GE Healthcare, Chalfont St Giles, Bucks, UK). Radiologists’ reports of patients’ chest radiographs (taken at the time of admission) were acquired from hospital records; we were interested in the presence of pleural effusions or atelectasis, which could cause reduced FRC. Length-of-stay data were acquired from hospital records.

10.1136/bmjresp-2017-000230.supp1 Supplementary file 1

### FRC, spirometry and cough measurements

The semirecumbent position was used for measurements as this replicates most stroke patients’ position in a hospital bed. It enabled us to compare cough flow results with those in our previously published study and include subjects who were unable to sit unsupported.^6^ A healthy control group was studied for comparison as normal values of FRC in the reclined position are not known.

FRC was measured using the closed circuit helium dilution technique on a Medisoft SpiroAir system controlled using a computer running Exp’Air software (Medisoft SA, Sorinnes, Belgium). The system consists of a 13 L rolling-seal spirometer with a katharometer-based helium analyser and a chemical fuel cell oxygen analyser. A Lilly-type cone pneumotachograph is incorporated. Participants sat in a supportive, reclinable chair (with a headrest and armrests) for testing (Barton I-400, Barton Medical Corp, Austin, Texas, USA). The chair back was reclined to 45° from upright and the legs raised to horizontal. Participants connected to the SpiroAir via a flanged mouthpiece (Hans Rudolf, Shawnee, Kansas, USA) and wore a nose clip. FRC testing was performed at least twice. The mean of two FRC values within 10% of each other was recorded. A minimum of 10 min interval was allowed between repeat tests to allow for helium washout. Total lung capacity (TLC), inspiratory capacity (IC) and slow vital capacity (VC) values were acquired by the participant making inspiratory and expiratory VC manoeuvres at the end of each FRC test cycle. Those able to sit and tolerate further tests then rested upright for 20 min, before FRC testing was repeated in the seated upright position. Patients unable to tolerate the reclined position, wearing the facemask, for the required time, had upright measurements only.

### Cough flow testing (using the rolling seal spirometer)

The rolling seal spirometer (as above) was set to measure a flow-volume loop. Participants were connected to the spirometer via a bacterial filter and an appropriately sized Hans-Rudolf facemask covering the nose, mouth and chin (Hans Rudolf). To ensure a good air seal, any gaps around the mask were filled with silicone nosepieces and Silly Putty, a mouldable mixture of silicone oil and boric acid (Binney & Smith Europe, Bedford, UK). The participant was asked to take a deep breath and cough as hard as possible into the mask. A minimum of three coughs was performed and the maximum flow value, out of three flows within 10% of each other, was recorded. Flows were recorded in litres per second and converted to the standard litres per minute by multiplying values by 60.

### Predicted values and statistical testing

FRC was expressed as per cent predicted (% predicted) to allow for differences in age, height or proportion of females between the two groups. VC, TLC and IC measurements were also recorded and expressed as % predicted. Cough flow rates were expressed as a percentage of predicted peak flow rate as there are no published normal ranges for cough peak flow in adults.

% predicted values were calculated using the European Community for Steel and Coal prediction equations.^26^ It was calculated by 31 participants in each group who would be required to find a difference of 9% predicted FRC between patients and controls based on an earlier study in chronic hemiplegics.^15^ An analysis of achieved power was performed after reclined FRC data had been obtained on 30 controls and 21 patients. It was calculated that the study had achieved 82% power at the 5% significance level so recruitment to the study was halted.

Datasets were tested for normality using the D’Agostino and Pearson omnibus method. Comparisons between patients and control groups were made using unpaired t-tests or Mann-Whitney U tests. Difference in proportions between groups was calculated using Fisher’s exact test. Where participants underwent FRC testing in both the reclined and seated positions, the results were compared using paired t-tests or Wilcoxon tests.

### Regression

Univariate regression analyses were performed to investigate predictors of the dependent variable, cough flow in the reclined position; all analyses used absolute (rather than % predicted) values of volume and flow and included a constant. Patients and controls able to complete FRC and cough flow tests in the reclined position were analysed together.

Univariate linear regression was also used on patient data to investigate the effect of FRC and cough flow on patient outcome (length of stay in hospital, dependent variable). All regression models included a constant. GraphPad Prism V.5.00 (GraphPad Software, San Diego, California, USA, www.graphpad.com) and SPSS Statistics V.17.0 computer software were used for statistical analysis and construction of figures. CIA software (University of Southampton, UK) was used to calculate CIs for non-parametric data.

## Results

The baseline characteristics of participants are given in table 1. Further details of stroke patients are given in table 2. Two patients were left-handed; both had left brain strokes.

**Table 1:** Baseline characteristics of participants

		Stroke patients	Controls	Difference (95% CI)	p Value
Total number assessed		27	30		
Age (years)	Mean (SD)	68 (11)	58 (11)	10 (4 to 16)	0.001*
Height (cm)		169.6 (7.9)	169.7 (12.2)	−0.1 (-5.6 to 5.6)	0.997*
Weight (kg)	Median (IQR)	78.0 (69.0 to 86.7)	72.1 (65.0 to 80.3)	4.2 (-3.0 to 11.0)	0.182†
Body mass index (kg/m^2^)		27.5 (24.0 to 30.0)	25.5 (23.0 to 29.0)	1.0 (4.0 to −1.0)	0.193†
O_2_ saturations breathing air (%)		97 (92 to 98)	97 (95 to 98)	0 (-1 to 1)	0.660†
Smoking (pack years)		0 (0 to 20)	0 (0 to 15)	0 (0 to 5)	0.485†
Ethnicity: white European/other (N)		21/6	28/2		
Proportion white European		0.78	0.93	−0.16 (−0.35 to 0.03)	0.138‡
Sex: males/females (N)		17/10	15/15		
Proportion male		0.63	0.50	0.13 (−0.12 to 0.36)	0.420‡
Smoking history: ever/never smoked (N)		13/14	12/18		
Proportion ever smoked		0.48	0.40	0.08 (−0.20 to 0.30)	0.599‡

*p Value calculated using unpaired t-test.

†p Value calculated using Mann-Whitney U test.

‡p Value calculated using Wilcoxon test.

**Table 2:** Stroke patient group details

Stroke severity on day of testing (National Institutes of Health Stroke Scale score, most severely impaired score 42)	Median (IQR)	4 (2 to 6)
	Range	0 to 21
Stroke lesion in left or right brain?	18 left brain 9 right brain	
	Proportion left brain	0.67
Thrombolysed with alteplase?	17 thrombolysed 10 not thrombolysed	
	Proportion thrombolysed	0.63
Number of days post stroke when tested	Median (IQR) in days	3 (2 to 6)
	Range in days	1 to 14
Total length of stay in hospital	Median (IQR) in days	9 (3 to 27)
	Range in days	2 to 78

For further explanation of the NIHSS see supplementary file 1.

**Figure 1:**
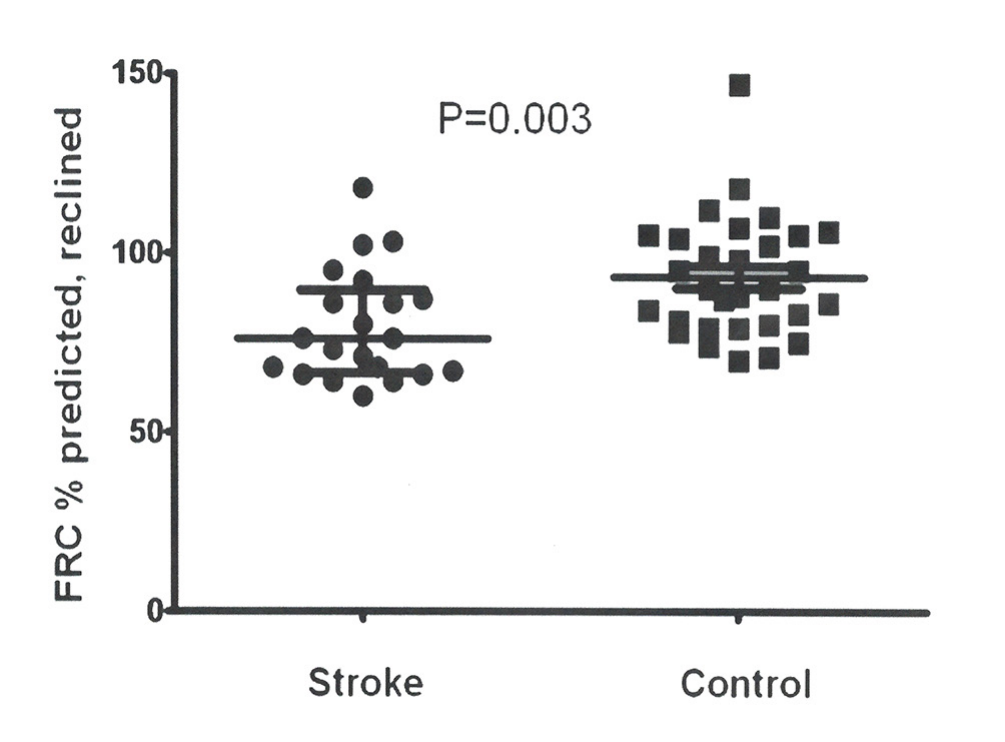
Comparison of reclined functional residual capacity (FRC) in stroke patients and controls. The figure shows FRC % predicted for 21 patients* and 30 controls. Medians and IQRs are marked with horizontal lines. See table 3 for further information. p Value calculated using a Mann-Whitney U test. *In total, 6 patients of the 27 assessed were unable to tolerate two FRC measurements in the reclined position.

**Figure 2:**
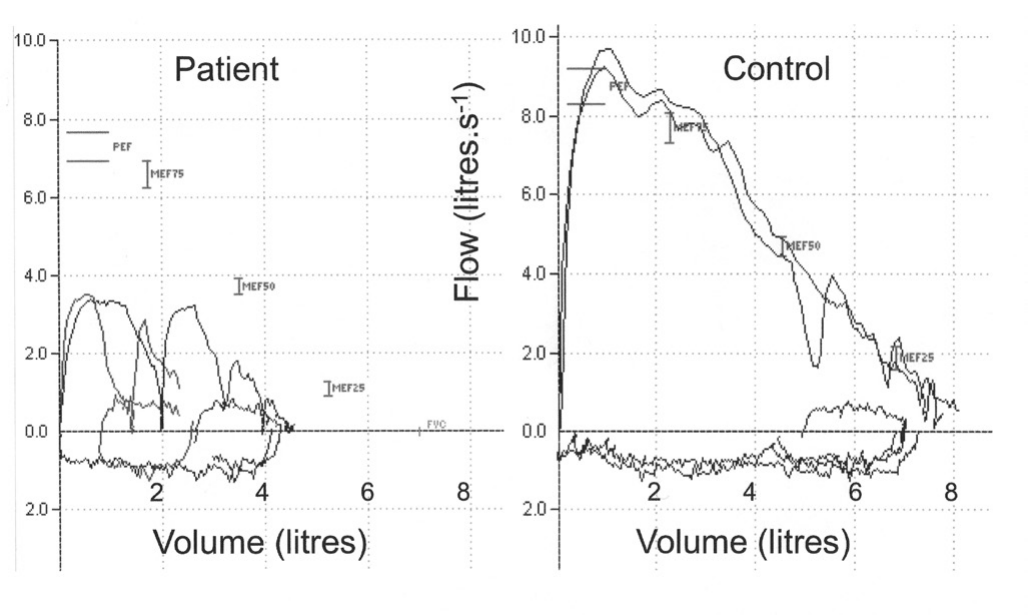
Cough flow traces on a flow-volume loop in a stroke patient and healthy control. The left panel shows coughs for a patient with a mild right hemiparesis. The right panel shows coughs for control. The patient’s trace shows reduced inspired and expired volume and cough flow compared with the control subject.

Twenty-seven patients had baseline assessments (table 1) and managed to complete cough flow and spirometry tests. Twenty-one managed to perform reclined FRC and other static lung volumes adequately (table 3, figure 1). Sixteen patients were able to perform both upright and reclined FRC. For a further five patients, only upright FRC was measured. Tests were limited by patients being unable to sit or lie still, in the required position, for the required length of time and by patients becoming excessively fatigued by the time and efforts required for the study. The results of cough flow tests and spirometry are given in table 4. Figure 2 shows the cough flows on a flow-volume loop for a patient and a control subject. The results of FRC performed in the upright seated position are given in table 5 together with data about the positional difference in FRC for those tested in reclined and seated positions.

**Table 3:** Functional residual capacity (FRC) and total lung capacity (TLC) in the reclined position

		Patients	Controls	Difference (95% CI)	p Value
	N	21*	30		
FRC (L)	Median (IQR)	2.50 (2.32 to 3.60)	2.78 (2.26 to 2.90)	−0.27 (−0.71 to 0.12)	0.162†
FRC (% predicted)		76.0 (66.5 to 89.5)	90.0 (79.8 to 105.0)	−14.0 (−22.0 to −5.0)	0.003†
TLC (L)	Mean (SD)	4.40 (0.37)	6.22 (0.28)	−1.83 (−2.75 to −0.92)	0.002‡
TLC (% predicted)		75.3 (16.0)	103.7 (12.4)	−28 (−37.3 to −19.6)	<0.001‡

*6 of 27 patients were unable to complete two reclined FRC measurements.

†p Value calculated using Mann-Whitney U test.

‡p Value calculated using Student’s t-test.

**Table 4:** Peak cough flow and spirometry in the reclined position

		Patients	Controls	Difference (95% CI)	p Value
	N	27	30		
Peak cough flow (L/min)	Mean (SD)	297 (133)	380 (121)	−83 (−153 to −14)	0.019
Peak cough flow (% pred PEF)		61.2 (32.6)	86.3 (17.3)	−25.1 (−38.8 to −11.4)	<0.001
Volume inspired before cough (L)		2.22 (0.83)	3.41 (0.72)	−1.19 (−1.72 to 0.67)	<0.001
Volume inspired before cough (% pred VC)		64.3 (19.5)	94.6 (15.6)	−30.1 (−42.2 to −18.5)	<0.001
Vital capacity (L)		2.75 (0.89)	4.06 (1.24)	−1.31 (−1.92 to −0.71)	<0.001
Vital capacity (% predicted)		79.1 (24.3)	111.9 (17.3)	−32.7 (−44.1 to −21.4)	<0.001
Inspiratory capacity (L)		2.47 (0.79)	3.56 (1.01)	−1.09 (−1.76 to −0.43)	0.002
Inspiratory capacity (% pred)		80.1 (19.5)	101.0 (13.7)	−20.8 (−33.3 to −8.4)	0.002

All p values calculated using Student’s t-test.

PEF, peak expiratory flow; VC, slow vital capacity.

**Table 5:** Functional residual capacity (FRC) values in the upright seated position and FRC change from reclined to upright

		Patients	Controls	p Value for difference between patient and control groups
	N	21*	30	
FRC seated (litres)	Median (IQR)	2.84 (2.30 to 3.15)	2.92 (2.54 to 3.84)	0.31†
FRC seated (% predicted)	Median (IQR)	84.0 (73.0 to 104.0)	99. 5 (86.5 to 112.3)	0.02†
	N	15*	30	
FRC change from reclined to upright (litres)	Median difference (IQR)	0.21 (0.1 to 0.37)	0.235 (–0.08 to 0.43)	0.88^†^
p Value for difference between paired (reclined and upright) values for each group		<0.01‡	<0.01‡	

*Of the 21 patients with upright FRC measurements, 6 did not have reclined FRC measurements, as they did not tolerate reclining for a prolonged period wearing the facemask; so there are 15 patients with paired upright and reclined measurements.

†p Value calculated using Mann-Whitney U test.

‡p Value calculated using Wilcoxon matched pairs test.

### Chest radiographs

Reports of chest radiographs were obtained for 14 patients, 11 of whom had an FRC measurement. For 8 of the 11, the lung fields were reported to be clear. One radiograph showed a small pleural effusion (patients’ FRC was 80% predicted); another showed linear atelectasis in the lower zones (patient FRC was 87% predicted). One further radiograph had patchy consolidation bilaterally (patient FRC 95% predicted). Complete details of the chest radiographs and the corresponding FRC results are shown in online supplementary file 1 and table S1 (within the additional file).

### Regression

#### Prediction of cough flow

**Figure 3:**
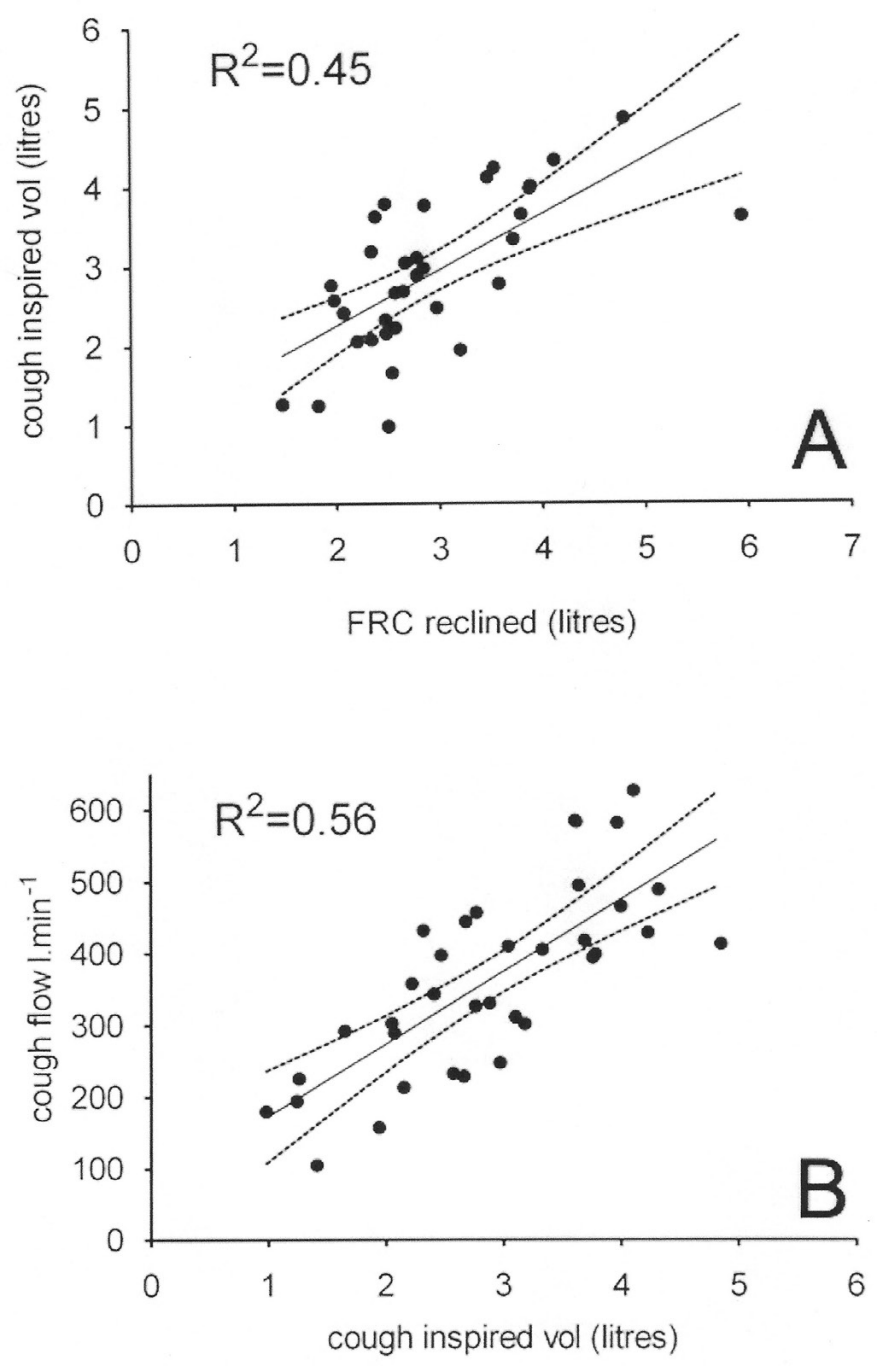
Reclined functional residual capacity (FRC) predicts cough inspired volume, which in turn predicts cough flow. All measures shown on the scatter plots were taken in the reclined position. (A) A scatter graph of FRC in 34 patients and controls plotted against cough inspired volume. (B) A scatter graph of cough inspired volume plotted against cough flow in 36 patients and controls. The regression line (solid) and its 95% CI (dotted lines) are marked on both panels.

Considering all participants together, volume inspired before cough (reclined) was the strongest predictor of reclined cough flow rate (adjusted R^2^=0.56, p<0.001). FRC (reclined) was a strong and significant predictor of volume inspired before cough (adjusted R^2^=0.45, p<0.01). See figure 3 for illustration. NIH stroke severity score was not a significant predictor of cough flow or FRC.

The regression equations are as follows: cough inspired volume (L)=−1.21 + 0.70 * FRC (L); adjusted R^2^=0.45 and p<0.01. 95% CI for the constant=−0.01 to 1.71% and 95% CI for the slope=0.42 to 0.98. Peak cough flow (L/min)=73 + 100*cough inspired volume (L); adjusted R^2^=0.56 and p<0.01. 95% CI for the constant=–19 to 165% and 95% CI for the slope=70 to 131. Age was not retained as a significant variable in regression analysis despite the difference in ages between patients and controls.

#### Prediction of length of hospital stay

A univariate linear regression analysis including only patients showed that reclined FRC (% predicted) was not a significant predictor of length of hospital stay (p=0.057). However, peak cough flow (% predicted PEF) was a significant predictor of length of hospital stay (R^2^=0.70, p<0.001).

## Discussion

This study is the first to measure FRC in patients <2 weeks after the onset of stroke. Patients’ mean FRC (% predicted) measured in the reclined position was significantly reduced compared with that of similar controls despite most patients having only mild overall stroke-related impairment. FRC was significantly related to peak cough flow; however, the most significant predictor of peak cough flow was the volume of air inspired prior to cough. The results show that despite good performance on a prognostic stroke scale patients can have significant respiratory impairment. Static (FRC, TLC) and dynamic (VC, IC, peak cough flow) lung volumes were all significantly reduced in patients.

### Critique of the method

The study includes a moderate number of patients and their stroke-related impairments were mostly mild. The median NIHSS score of 4 on the day of respiratory testing means an excellent outcome is likely.^25^ There was a narrow range of stroke severity scores and this may be the reason that stroke severity did not emerge as a significant predictor of FRC or of peak cough flow, as it did in our previous study.^6^ Patients were excluded if they could not consent, follow commands or if they could not use the mouthpiece correctly: patients with cognitive defects after stroke have been shown to be more likely to aspirate^7^ and we speculate that those excluded may have had even lower lung volumes than the included patients. The moderate numbers meant it was not possible to compare those with left and right brain strokes but we do not believe this would add to the clinical usefulness of our study. Ideally, further experiments would be performed to determine if and how FRC changes with stroke recovery.

Deep inspiration and coughing during testing could lead to expansion of areas of pulmonary atelectasis and an increase in FRC. However, this was the case for both patients and controls. Sitting FRC was measured for 16 patients and 30 controls. This was a secondary measure performed after the end of reclined testing. The increase shown in patients’ mean FRC on sitting reflects what happens for the least affected patients; the five worst affected were unable to complete upright FRC tests.

Three published studies describe FRC in chronic hemiparetic stroke but they include only small numbers and have conflicting findings. Studies by Cohen (seven patients, FRC mean (SD) 101 (22) % predicted) and Lanini (eight patients, FRC 99 (17) % predicted) found no reduction in patients’ FRC measured by helium dilution in the seated position.^13 18^ Fugl-Meyer studied FRC (also by helium dilution) in 54 patients and 12 controls lying flat. For 27 severely stroke-affected (FRC 69 (26) % predicted) and 18 markedly affected patients (FRC 82 (12) % predicted) FRC was significantly reduced compared with 12 controls (FRC 83 (9) % predicted); p<0.05 for both. For nine mildly affected patients, FRC (74 (15) % predicted) was not significantly reduced compared with the controls.^15^

The FRC we found for 21 acute stroke patients in our study (median reclined FRC 76% predicted, IQR 67% to 90%) was very similar to that found by Fugl-Meyer for a smaller group of chronic stroke patients with mild to moderate impairments.^15^ Peak cough flow for the patients (mean 297 L/min, SD 133) was in line with that found in a recently work published by our group (mean 287 L/min, SD 171) and others (mean 262, SD 188).^6 7^

It is proposed that the reduced FRC as well as TLC, VC and IC after cortical stroke are due to several factors. These include weak respiratory muscles, at least when acting under voluntary control, and consequent displacement of the diaphragm cranially in addition to decreased expansion and convexity of the chest wall on the hemiparetic side. Reduced FRC in the patients compared with controls is unlikely to be due to patients’ slightly higher body mass index; the median difference between the two groups was only 1 kg/m^2^ (95% CI 4 to −1 kg/m^2^) and the difference was not statistically significant. The patients’ low FRC is unlikely to be due to atelectasis. Of the chest radiographs retrieved for 11 stroke patients, 7 were reported as normal and the median reclined FRC of this subgroup was similar to that of the whole group. Increased elastic recoil of the lungs is very unlikely to be the reason for decreased FRC as all patients were free of lung disease and stroke is not known to affect lung parenchyma directly.

Lastly we did not assess mental state directly although components are within the NIHSS, but all participants could understand the consent process and so we doubt if this influenced our findings.

Low peak cough flow rate is associated with an increased risk of chest infections and possibly death in stroke patients.^3–5^ FRC was reduced in patients at an early stage post stroke despite only mild stroke-related impairments.

The volume of air inspired prior to cough was the modifiable predictor accounting for most of the variability in peak cough flow, with an adjusted R^2^ of 0.56 (p<0.001).

Moving stroke patients from supine to upright significantly increases the FRC. The median increase in FRC for 15 patients was 210 (95% CI 100 to 370) mL and this repositioning was only from 45° reclined to upright. Intermittent application of continuous positive airway pressure may also increase end expiratory lung volume. Both strategies are suitable for patients unable to cooperate due to lack of understanding and motor control secondary to stroke.

Increasing inspired volume prior to cough would seem a logical strategy to try and increase peak cough flow in stroke patients. Asking patients to try and increase their own inspired volume by verbal instruction and demonstration is simple and may promote recovery of the cough motor control in the damaged or contralateral cerebral hemisphere, which seems unlikely to occur with passive therapies such as use of mechanical insufflation-exsufflation devices (eg, CoughAssist, Philips-Respironics, Andover, Massachusetts, USA). Early intervention after acute stroke may prevent chronically reduced FRC and reduce morbidity and mortality. This work is important since numbers of stroke survivors are increasing and so are the number of patients suffering with respiratory complications.

## Conclusions

FRC (% predicted) measured in the reclined position is significantly reduced in acute hemiparetic stroke patients compared with controls; this is associated with poor cough flow. Interventions which increase FRC deserve evaluation in middle cerebral artery (MCA) stroke patients at risk of chest infection.
