# Supplementary material for: Poor cough flow in acute stroke patients is associated with reduced functional residual capacity and low cough inspired volume
Source: BMJ Open Respir Res. 2017 Oct 26;4(1):e000230. doi: 10.1136/bmjresp-2017-000230 (PMC5663270; doi:10.1136/bmjresp-2017-000230)
Supplement: Supplementary file 1 [file bmjresp-2017-000230supp001.pdf]

POOR COUGH FLOW IN ACUTE STROKE PATIENTS IS ASSOCIATED WITH REDUCED FUNCTIONAL RESIDUAL CAPACITY AND LOW COUGH INSPIRED VOLUME

Supplementary file 1

Additional methods

Assessment of stroke severity

Patients' stroke severity was assessed using the National Institute for Health Stroke Scale (NIHSS)[S1, S2]. The NIHSS is a 15-item neurologic examination stroke scale used to evaluate the effect of acute cerebral infarction on the levels of consciousness, language, neglect, visual-field loss, extraocular movement, motor strength, ataxia, dysarthria, and sensory loss. A trained observer rates the patient's ability to answer questions and perform activities. Ratings for each item are scored with 3 to 5 grades with 0 as normal, and there is an allowance for un-testable items. It was designed for use in trials of stroke therapy and shown to be quick and easy to administer and valid as well as having high inter-rater reliability and high test-retest reliability[S2]. It first came to be widely used in trials of thrombolysis in the 1990s and has become part of the initial and ongoing assessment of all stroke patients being considered for thrombolysis. The maximum score is 42 reflecting the maximum disability; the minimum score is 0. A score of 16 or more forecasts a high probability of death or severe disability whereas a score of 6 or less predicts a good recovery[S3].

Additional Results

Chest radiographs

Reports of chest radiographs were obtained for fourteen patients, eleven of whom had an FRC measurement. See Table S1.

Table S1 Reclined FRC values and chest radiograph reports for stroke patients

The results are for 11 patients for whom reports of chest radiographs were available. This research was performed at a tertiary stroke centre. Patients without chest radiographs had been transferred in after initial assessments at district general hospitals.

FRC % predicted, reclined position	Chest radiograph report
67	Lungs clear
68	Normal
71	Clear
73	Clear
76	Lungs clear
80	Small right pleural effusion
86	Clear lung fields
87	Linear atelectasis left lower zone
92	Normal
95	Patchy consolidation left and right
118	Hyperinflated

Key for Table A1

FRC=functional residual capacity; %=percentage

References

S1. National Institute for Health, National Institute for Neurological Disorders and Stroke. The NIH Stroke Scale: US Government,; 1989 [Available from: https://stroke.nih.gov/resources/scale.htm.

S2. Brott T, Adams HP, Jr., Olinger CP, et al. Measurements of acute cerebral infarction: a clinical examination scale. Stroke. 1989;20(7):864-70.

S3. Adams HP, Jr., Davis PH, Leira EC, et al. Baseline NIH Stroke Scale score strongly predicts outcome after stroke: A report of the Trial of Org 10172 in Acute Stroke Treatment (TOAST). Neurology. 1999;53(1):126-.
